# Implications of aging in the treatment of complex arrhythmias

**DOI:** 10.18632/aging.101620

**Published:** 2018-10-27

**Authors:** Sofia Chatzidou, Georgios Georgiopoulos, Christos Kontogiannis

**Affiliations:** 1 Department of Clinical Therapeutics, “Alexandra” Hospital, University of Athens, Athens, Greece

**Keywords:** electrical storm, aging, ventricular arrhythmias, β-blockers, adrenergic receptors

The failing myocardium is characterized by numerous inter-related perturbations at molecular, cell and tissue level. Notably, aged myocardium and heart failure (HF) share common pathophysiologic processes and accumulating evidence suggests that impairment of sympathetic nervous system may modulate the clinical course of both entities. Within the sympathetic nervous system, adrenergic receptors (ARs) and changes in regulation of their responses are considered the connecting link for both aging and cardiovascular disease. In particular, decreased β-AR responsiveness, increased circulating catecholamines, and overall hyposensitivity to adrenergic stress are common pathophysiologic derangements in cardiovascular aging and HF [1].

We have previously shown that oral propranolol is more efficient and faster than metoprolol in the treatment of patients with implantable cardioverter-defibrillator (ICD) who experience an electrical storm (ES). This was the first study to compare the efficacy of a non-selective (propranolol) versus a selective β-blocker (metoprolol) towards the management of complex ventricular arrhythmias in HF patients [2].

It is well known that the failing human heart activates compensatory mechanisms, among which increased sympathetic activity has a key role on effector signaling and downstream targets. Adrenergic receptors are divided in two classes: α and β. In terms of β-ΑRs, β1-AR is primarily located in the myocardium while β2-AR is widely distributed in multiple tissues and β3-AR is found in adipose tissue [3]. As further explained in the specific study, failing myocardium exhibits a progressive decrease in β1/β2 ratio that is attributed to selective down regulation of the β1 receptors. Propranolol may effectively block signal transductions from both β1 and -unblocked from metoprolol- upregulated β2 cardiac receptors. Given that HF patients are also characterized by increased age, it is tempting to speculate that the combined effect of myocardium disease and aging inflates β-AR stimulation and ES represents an extreme form of sympathetic overdrive that necessitates a more potent β-blocker for prompt and successful treatment. Along this line, protein kinase A (PKA) is an important mediator of signal transduction downstream of G-protein coupled receptors in response to cAMP. Protein kinase A is comprised of two regulatory subunits, RI and RII. As PKA (and in specific, RIIβ) declines with aging, the β-adrenergic pathway is activated to higher equilibrium [4]. On the other hand, activated β-AR signaling has also been implicated in the failing heart. Indeed, preclinical data have demonstrated that loss of the regulatory RIIβ subunit confers survival benefits and resistance to age-related conditions including cardiac failure [4]. Collectively, increased β-AR activity in aged patients with HF who sustain an ES entails the use of most potent β-blocker. Despite the fact that selective β-blockers can also inhibit β2-ARs at high levels, clinical practice dosage of propranolol is superior to metoprolol. Of note, propranolol also exerts an anesthetic-like membrane action that might favorably affect the treatment of recalcitrant arrhythmias.

It should also be acknowledged that aging modifies the arrhythmogenic substrate beyond β-AR desensitization. Alterations in intrinsic electrical properties including the action potential duration, the effective refractory period and slower conduction in the atrioventricular junction, myocardium fibrosis, atria enlargement, amyloid infiltration, increased scarring in grounds of ischemia and elevated stretch and perpetual electrical and structural remodeling precipitate recurrent arrhythmias and hamper the response to anti-arrhythmic drugs [5]. Henceforward, selective β-blockers might not be as effective as non-selective, potent β-blockers in these patients under settings of malignant arrhythmias.

Another aspect that should be taken into consideration when comparing propranolol versus metoprolol in the treatment of ES is related to pharmacokinetic changes that are prevalent in older subjects. In this context, bioavailability of drugs that undergo a high rate of first-pass metabolism may be increased in elderly patients due to impaired first-pass extraction. As a matter of fact, propranolol is characterized by extensive high first-pass metabolism by the liver, resulting in 25% of propranolol to be detected in the systemic circulation as compared to 50% first-pass metabolism for metoprolol. In addition, propranolol is highly lipophilic and the majority (~90%) of propranolol is bound to plasma proteins (mainly albumin). Importantly, propranolol also binds to α1 Acid glycoprotein (AAG) and AAG increases with age [6] leading to an elevation in the free fraction of the active drug. Moreover, decrease in total water content in elderly and in albumin levels further yields an increased volume of distribution (Vd) of lipid soluble propranolol. Finally, Glomerular filtration rate (GFR) gradually declines with age and in combination with impaired oxidation capacity of elderly precipitates reduced clearance of propranolol. On the contrary, only a small fraction of metoprolol is bound to human serum albumin and no significant differences in systemic availability of the drug have been reported in patients with renal failure [7,8]. Overall, the pharmacokinetic properties of propranolol in elderly patients with ES favor more rapid achievement of therapeutic levels of this β-blocker with oral administration in respect to metoprolol.

In summary, the results obtained by our group on the relative efficacy of oral propranolol over metoprolol for management of ES, might partially be attributed to inherent differences in pharmacokinetic properties and pharmacodynamic responses, conditional to β-ARs number and affinity as well as signal transduction mechanisms in the failing myocardium. Importantly, age-related changes in sympathetic system signaling lie in parallel with pathophysiologic processes of HF and may further magnify therapeutic differences between selective and non-selective β-blockers in treatment of arrhythmias. Future research is warranted to address if sympathetic system dysfunction in the aged constitutes a primary disease or stems from regulatory mechanisms of cardiac decline and to evaluate therapeutic implications in clinically relevant populations of elderly patients.
